# Improved Schmidt Conversion of Aldehydes to Nitriles Using Azidotrimethylsilane in 1,1,1,3,3,3-Hexafluoro-2-propanol

**DOI:** 10.3390/molecules21010045

**Published:** 2015-12-29

**Authors:** Hashim F. Motiwala, Qin Yin, Jeffrey Aubé

**Affiliations:** Department of Medicinal Chemistry, Delbert M. Shankel Structural Biology Center, University of Kansas, 2034 Becker Drive, West Campus, Lawrence, KS 66047, USA; hashimmotiwala@ku.edu (H.F.M.); qinyin@ku.edu (Q.Y.)

**Keywords:** Schmidt reaction, aldehydes, nitriles, HFIP

## Abstract

The Schmidt reaction of aromatic aldehydes using a substoichiometric amount (40 mol %) of triflic acid is described. Low catalyst loading was enabled by a strong hydrogen-bond-donating solvent hexafluoro-2-propanol (HFIP). This improved protocol tolerates a broad scope of aldehydes with diverse functional groups and the corresponding nitriles were obtained in good to high yields without the need for aqueous work up.

## 1. Introduction

**Figure 1:**
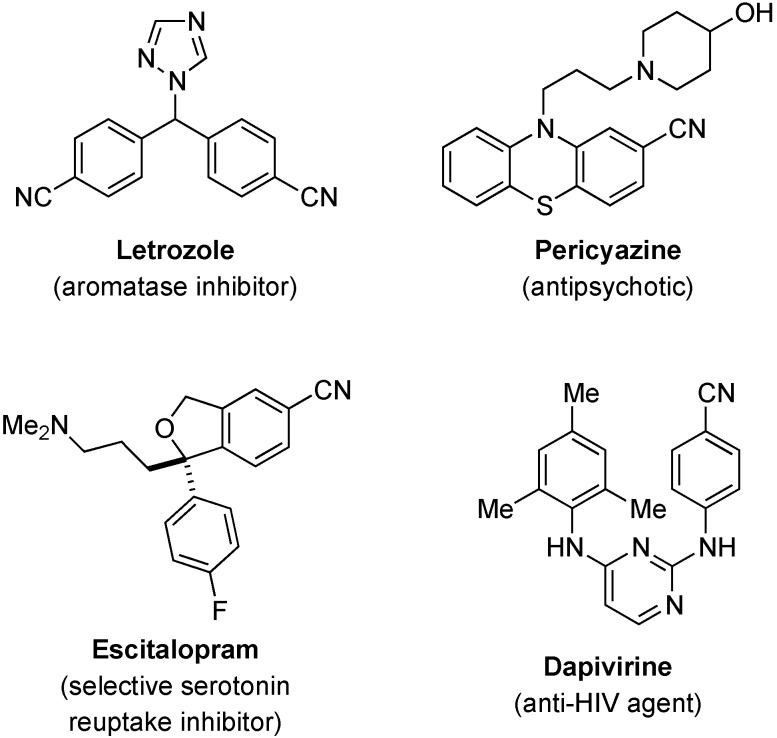
Drugs containing aromatic nitriles.

Nitriles are versatile building blocks and precursors to other functionalities such as acids, amines, amides, aldehydes, and tetrazoles. In addition, they are important structural motifs in many natural products [1], pharmaceuticals [2], agrochemicals, and dyes [3,4,5,6]. Aromatic nitriles are particularly well-represented in pharmaceutical agents, such as those depicted in Figure 1 [2]. Nitrile groups on the aromatic ring have been viewed as ketone bioisosteres and may increase resistance of aromatic system to the oxidative metabolism [2].

General strategies for the synthesis of aromatic nitriles include the Sandmeyer reaction of aryldiazonium salts [6,7,8,9], Rosenmund–von Braun reaction from aryl halides [9,10,11], transition metal-catalyzed cyanation of aryl halides [12,13,14,15] or direct cyanation through C–H bond functionalization of arenes [16,17,18,19,20], and ammoxidation of methyl arenes, which is a preferred industrial process [21,22,23]. Major drawbacks for most of these processes are the use of stoichiometric to excess amounts of toxic cyanide source, generation of heavy metal waste, requirement of relatively high temperatures (often >100 °C), long reaction times, or the requirement of a reactive aryl halide source (aryl iodides and bromides are generally preferred) [14,24]. Recently, other approaches, such as the dehydration of primary amides [25,26,27,28] or aldoximes [29,30,31,32], and one pot synthesis from aldehydes [33,34,35,36,37,38,39,40,41,42,43] have gained particular attention in lieu of directly attaching the nitrile group. However, harsh reaction conditions, high temperatures, and functional groups intolerance are some of the problems still associated with these recent methods. 

**Scheme 1:**
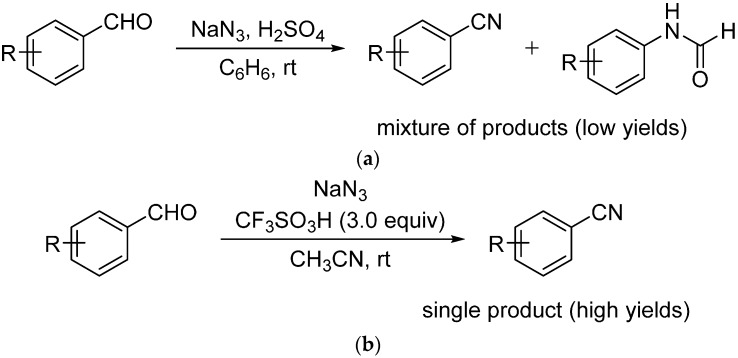
Schmidt Reactions of Aromatic Aldehydes. (**a**) Classical Schmidt reaction of aromatic aldehydes (McEwen; [45]); (**b**) Chemoselective Schmidt reaction of aldehydes to nitriles (Prabhu; [46]).

An attractive alternative to the above methods is the Schmidt reaction of aromatic aldehydes with hydrazoic acid as in principle it can deliver the nitriles in one straightforward step [44]. However, historically this reaction has provided a mixture of nitriles and formylanilides (Scheme 1a), thus limiting its utility [45]. Recently, Prabhu and co-workers demonstrated that the Schmidt reaction of aldehydes with sodium azide (NaN_3_) in the presence of triflic acid (TfOH) as a catalyst and acetonitrile (ACN, CH_3_CN) as solvent exclusively affords the corresponding nitriles (Scheme 1b) [46]. In order to achieve complete conversions, 3 equiv of TfOH was minimally required for high yields of the aromatic nitriles. For example, only 6% conversion was observed when 1.5 equiv of TfOH was used during their optimization studies [46]. Similarly, good results can be obtained using a catalyst in an ionic liquid medium [47]. A one-pot sequential Schmidt/Ritter reactions in the presence of 4 equiv of HBF_4_**·**OEt_2_ (2 equiv for each reaction) was also reported for the synthesis of *N*-*tert*-butylbenzamides from benzaldehydes [48]. We recently reported an efficient substoichiometric catalytic version of another type of Schmidt reaction, specifically the intramolecular Schmidt reaction of ketones with alkyl azides. In that chemistry, using 1,1,1,3,3,3-hexafluoro-2-propanol (HFIP, (CF_3_)_2_CHOH) was key to high yields using low loadings of HCl generated *in situ* from dissolving acetyl chloride in the solvent [49]. These results prompted us to investigate the strong hydrogen bond donor ability of HFIP in the intermolecular Schmidt reaction of aromatic aldehydes.

## 2. Results and Discussion

### 2.1. Optimization of Reaction Conditions

As reported by Prabhu [46], we began our studies on the reaction of 4-nitrobenzaldehyde **1a** with NaN_3_ and TfOH, replacing ACN as reported by Prabhu with HFIP (Table 1, entry 1). Low conversions of **2a** with 50 mol % TfOH (entry 1) and 80 mol % AcCl (entry 2) were obtained from these experiments, likely resulting from the low solubility of NaN_3_ in HFIP. Changing to azidotrimethylsilane (TMSN_3_) as a soluble azide source drastically improved the yield with 25 mol % of acid catalysts (entries 3 and 4). However, incomplete reactions accompanied by polar byproducts were still observed (TLC) despite long periods of stirring. Both AcCl and TiCl_4_ are converted to HCl when dissolved in HFIP, so the comparable results seen in entries 2 and 3 make sense taking into account the fact that TiCl_4_ provides fourfold more acid than AcCl. We therefore returned to using triflic acid with TMSN_3_ as the azide source. Even though the reaction with 30 mol % TfOH offered complete conversion in 2 h, only a modest yield of nitrile was obtained, again with unidentified byproducts (entry 5). Gratifyingly, a 1:1 solvent combination of HFIP and ACN significantly increased the yield but complete conversion was not achieved even after 4 h (entry 6). Finally, the reaction of **1a** with 40 mol % TfOH in HFIP/ACN (1:1) mixture proved optimal, providing a slightly better yield of **2a** along with a much shorter reaction time (entry 7).

**Table 1:** Optimization of the Schmidt Reaction of 4-Nitrobenzaldehyde **1a** ^a,b^. 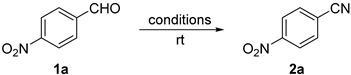

Entry	Azide Source	Azide (equiv)	Catalyst	Catalyst (mol %)	Solvent	Time (h)	NMR Ratio (2a:1a) ^c^	Yield (%) ^d^ 2a
1	NaN_3_	1.5	CF_3_SO_3_H	50	HFIP	16	30:70 ^e^	ND
2	NaN_3_	1.5	CH_3_COCl ^f^	80	HFIP	8	19:81	ND
3	TMSN_3_	1.5	TiCl_4_^g^	25	HFIP	24	ND	75
4	TMSN_3_	1.5	CF_3_SO_3_H	25	HFIP	8	ND	68
5	TMSN_3_	2.0	CF_3_SO_3_H	30	HFIP	2	ND	65 ^h^
6	TMSN_3_	2.0	CF_3_SO_3_H	30	HFIP/ACN (1:1)	4	ND	81
**7**	**TMSN_3_**	**2.0**	**CF_3_SO_3_H**	**40**	**HFIP/ACN (1:1)**	**45 min**	**ND**	**83**

^a^ To a solution of 4-nitrobenzaldehyde **1a** (0.25 or 0.50 mmol) and azide in solvent (0.50, 1.0, or 2.0 mL) was added a catalyst and the reaction was allowed to stir at rt for a specified period. ^b^ Concentration of **1a** was *ca.* 0.25 or 0.50 M. ^c 1^H-NMR ratio was determined on a crude reaction mixture. ^d^ Corrected isolated yield of **2a** (**2a** was contaminated with a small amount (*ca.* 3%–6%) of **1a**). ^e^ Other byproducts were also observed. ^f^ Could generate 80 mol % HCl *in situ*. ^g^ A 1.0 M solution of TiCl_4_ in CH_2_Cl_2_ was used. ^h 1^H-NMR only showed peaks of **2a**. ND = Not determined.

### 2.2. Substrate Scope

A series of aromatic aldehydes was examined under the optimized reaction conditions (Table 2). A wide array of functional groups on the aldehydes was well tolerated and the corresponding nitriles were obtained in good to excellent yields. Benzaldehydes containing electron-withdrawing substituents at the para position gave the corresponding nitriles in good yields (entries 1–5). Benzaldehyde **1e** required a slightly higher catalyst loading (60 mol %) to achieve a good conversion of the nitrile **2e** (entry 5). Electron-rich substrates with a broad range of functional groups such as hydroxyl, *O*-allyl, and *O*-propargyl at the para position underwent facile conversion (entries 6–14). Due to the presence of a basic amine, the substrate with a morpholine substituent needed 1.4 equiv of triflic acid, where 1.0 equiv of acid probably ended up in the amine salt (entry 13). Biphenyl substrate **1o** afforded nitrile **2o** in 80% yield (entry 15). The resulting nitriles were obtained in slightly lower yields for the *meta*- and *ortho*-substituted benzaldehydes (entries 16–18). Disubstituted benzaldehydes were also efficiently converted to the desired nitriles in good to high yields (entries 19–25). 2-Naphthonitrile **2z** was readily prepared in 77% yield from 2-naphthaldehyde **1z** (entry 26). The scope could be extended to heteroaromatic aldehydes affording the representative nitriles in good yields (entries 27 and 28). Throughout, we found that the position of the substituents on the phenyl ring had a relatively minimal influence on the reaction outcome.

**Table 2:** Scope of Aromatic Aldehydes ^a,b^. 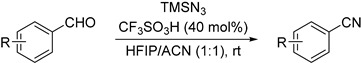

Entry	Aldehyde 1	Nitrile 2 (% yield) ^c^	Entry	Aldehyde 1	Nitrile 2 (% yield) ^c^
1	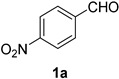	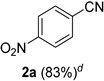	17	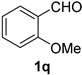	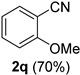
2	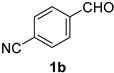	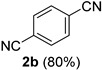	18	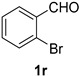	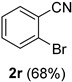
3	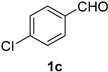	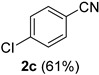	19	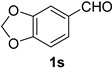	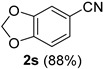
4	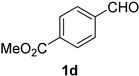	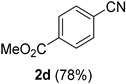	20	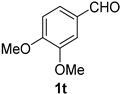	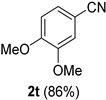
5	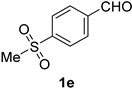	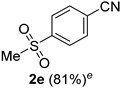	21	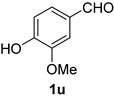	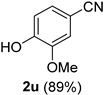
6	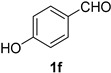	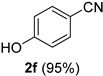	22	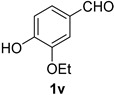	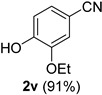
7	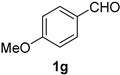	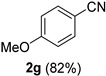	23	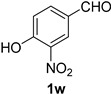	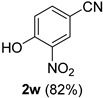
8	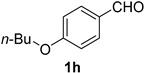	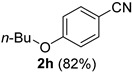	24	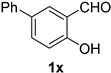	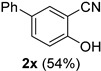
9	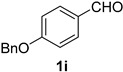	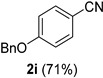	25	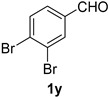	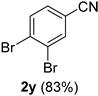
10	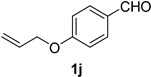	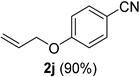	26	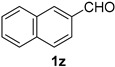	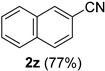
11	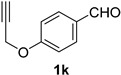	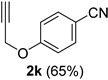	27	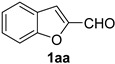	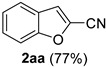
12	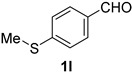	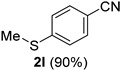	28	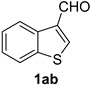	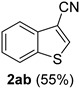
13	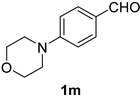	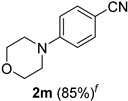	29	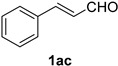	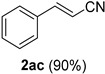
14	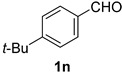	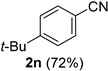	30	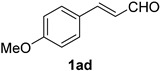	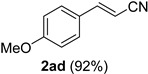
15	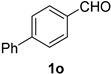	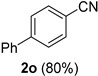	31	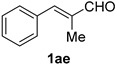	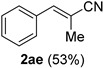
16	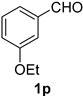	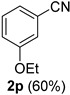	32	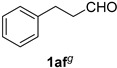	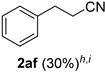

^a^ To a solution of aldehyde **1** (1.0 equiv) and TMSN_3_ (2.0 equiv) in a premixed HFIP/ACN solvent mixture (2.0 mL, 1:1) was added TfOH (40 mol %) and the reaction was allowed to stir at rt for a period of 20–75 min. ^b^ Concentration of aldehyde **1** was *ca.* 0.25 M. ^c^ Isolated yields. ^d^ Contains *ca.* 4% of unreacted **1a** (see the Experimental Section for details). ^e^ TfOH (60 mol %) was used. ^f^ TfOH (1.4 equiv) was used. *^g^* Commercially used **1af** was *ca.* 77% pure. ^h^ TfOH (25 mol %) and TMSN_3_ (3.0 equiv) was used; see the Experimental Section for details. ^i^ Yield of **2af** was not corrected w.r.t. 77% purity of **1af**.

Cinnamaldehydes **1ac** and **1ad** lacking double bond substitution reacted smoothly to afford the resultant cinnamonitriles in excellent yields (entries 29–30) whereas α-methyl substituted cinnamaldehyde **1ae** provided the nitrile **2ae** in only 53% yield (entry 31). We would have been pleased if this method were extendable to aliphatic ketones, which have proved problematic in previous methods as well. Unfortunately, reaction of an aliphatic aldehyde, hydrocinnamaldehyde **1af**, with 3 equiv of TMSN_3_ in the presence of 25 mol % TfOH resulted in a complex mixture from which 3-phenylpropionitrile **2af** was isolated in low yield (entry 32). Accordingly, additional aliphatic aldehydes were not explored.

**Scheme 2:**
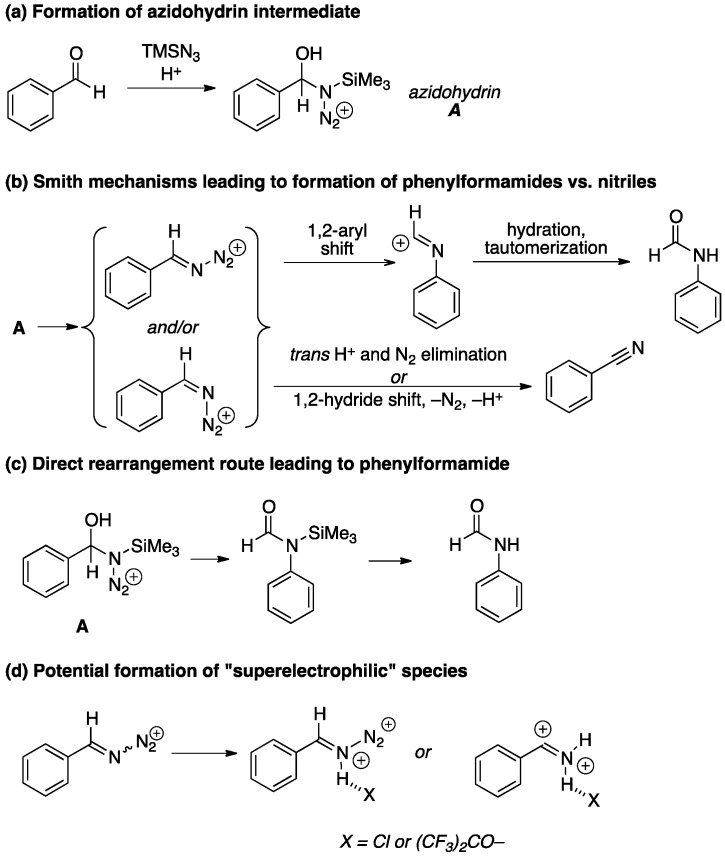
Mechanistic possibilities. In all cases, the SiMe_3_ group might be replaced by H under the reaction conditions (leading to exactly analogous pathways).

This seemingly simple transformation raises a number of interesting mechanistic questions (Scheme 2). Most workers have adopted some variation of the mechanism originally suggested by P. A. S. Smith [50], in which an initially formed azidohydrin adduct **A** loses water to afford a pair of equilibrating diazoiminium ions, which can undergo migration of the phenyl group leading to phenylformamide after re-hydration and tautomerization (Scheme 2b). Alternatively, hydride migration followed by deprotonation would similarly afford nitrile; a variation that involves the same intermediate would entail an E2-style elimination of a proton and nitrogen gas, although this is rarely proposed. Confining oneself to the Smith manifold in Scheme 2b, it is hard to justify why a change in solvent would effect the essentially exclusive formation of nitrile since that would most likely be a matter of either intrinsic migration potential between a phenyl *vs.* hydride or differences in the ratio of the acyliminium ion stereoisomers shown in brackets (in general, the barrier for the interconversion between these is thought to be high) [51]. On the other hand, Ostrovskii *et al.* have suggested that the Smith dehydration mechanism, leading to nitrile, is in competition with a direct rearrangement pathway, leading to phenylformamide (Scheme 2c) [52,53]. Acidic HFIP is a strongly dehydrating medium, which would be consistent with this observation. Finally, it is tempting to speculate that “superelectrophilic” species [54] like the protonated (or hydrogen bonded) diazoiminium ion or nitrilium ions shown in Scheme 2d might also be involved, although this must remain, for the moment, an intriguing conjecture pending further mechanistic work.

## 3. Experimental Section

### 3.1. General Information

Reactions were performed in glass sample vial with rubber lined cap. All chemicals were used as received from commercial source, without further purification. Acetonitrile was dried by passage through neutral alumina columns using a commercial solvent purification system prior to use. Thin-layer chromatography (TLC) was performed using commercial glass-backed silica plates (250 microns) with an organic binder. Visualization was accomplished with UV light. Flash chromatography was carried out on a CombiFlash^®^ purification system using a 4 g normal phase silica flash column. Infrared (IR) spectra were acquired as a solid (Shimadzu FTIR-8400S, Kyoto, Japan). All nuclear magnetic resonance (NMR) spectra (^1^H, ^13^C, APT) were recorded on a 400 MHz instrument (Bruker AV-400, Billerica, MA, USA). NMR spectra were recorded in deuterated chloroform. Chemical shifts are reported in parts per million (ppm) and are referenced to the center line of the solvent (δ 7.26 ppm for ^1^H-NMR and δ 77.23 for ^13^C-NMR, respectively). Coupling constants are given in Hertz (Hz). Melting points were determined on an automated melting point apparatus and are uncorrected. A sample concentrator using N_2_ gas was used for the concentration of reaction mixtures. Spectroscopic data for the aromatic nitriles prepared according to the methodology described in this paper matched well with those reported in the literature.

### 3.2. General Procedure for the Optimization of Reaction Conditions for the Synthesis of 4-Nitrobenzonitrile ***2a***

To a solution of 4-nitrobenzaldehyde **1a** (0.25 or 0.50 mmol, 1.0 equiv) and NaN_3_ or TMSN_3_ (1.5–2.0 equiv) in HFIP or HFIP/ACN mixture (0.50, 1.0, or 2.0 mL) was added a catalyst (effervescence due to nitrogen gas evolution was immediately observed). The vial was capped and the reaction mixture was allowed to stir at rt for a specified period (45 min to 24 h). The reaction mixture was concentrated under nitrogen. The residue obtained was diluted with appropriate solvent (CH_2_Cl_2_ or EtOAc) and was either filtered through a Pasteur pipette containing a cotton plug to get a crude ^1^H-NMR ratio (for entries 1 and 2) or purified using a 4 or 12 g normal phase silica flash column on a CombiFlash purification system with a gradient elution of 0%–10% EtOAc/hexanes (for entries 3–7). Concentration of the appropriate fractions afforded 4-nitrobenzonitrile **2a** contaminated with a small amount (*ca.* 3%–6%) of **1a** (except for entry 5, where pure **2a** was obtained).

### 3.3. General Procedure A for the Synthesis of Aromatic Nitriles

To a solution of an aromatic aldehyde **1** (0.500 mmol, 1.0 equiv) and TMSN_3_ (115 mg, 1.00 mmol, 2.0 equiv) in a premixed HFIP/ACN mixture (2.0 mL, 1:1) in a nitrogen-flushed two dram vial was added triflic acid (TfOH; 17.7 μL, 0.200 mmol, 0.40 equiv) (exotherm and brisk effervescence due to nitrogen gas evolution was immediately observed). The vial was capped and the reaction mixture was allowed to stir at rt for 20–75 min. The reaction mixture was concentrated under nitrogen. The residue obtained was suspended in CH_2_Cl_2_/hexanes mixture and loaded on a silica gel in a 5 g sample cartridge. Purification using a normal phase silica flash column on a CombiFlash purification system afforded a corresponding aromatic nitrile **2** upon concentration of appropriate fractions.

*4-Nitrobenzonitrile* (**2a**) [46]: Following the general procedure **A**, a solution of 4-nitrobenzaldehyde **1a** (75.6 mg, 0.500 mmol, 1.0 equiv) and TMSN_3_ (115 mg, 1.00 mmol, 2.0 equiv) in HFIP/ACN mixture (2.0 mL, 1:1) was treated with TfOH (17.7 μL, 0.200 mmol, 0.40 equiv). The reaction mixture was stirred at rt for 45 min. Purification using a 4 g flash column on a CombiFlash purification system (0%–10% EtOAc/hexanes over 40 min) afforded **2a** along with a small amount of unreacted **1a** (eluted between 2.3%–4.0% EtOAc/hexanes) as a colorless crystalline solid (61.6 mg, 0.416 mmol, 83% corrected yield; contains *ca.* 4% of **1a** as determined by ^1^H-NMR).

*Terephthalonitrile* (**2b**) [55]: Following the general procedure **A**, a solution of 4-cyanobenzaldehyde **1b** (65.6 mg, 0.500 mmol, 1.0 equiv) and TMSN_3_ (115 mg, 1.00 mmol, 2.0 equiv) in HFIP/ACN mixture (2.0 mL, 1:1) was treated with TfOH (17.7 μL, 0.200 mmol, 0.40 equiv). The reaction mixture was stirred at rt for 60 min. Purification using a 4 g flash column on a CombiFlash purification system (0%–10% EtOAc/hexanes over 40 min) afforded **2b** (eluted between 5.0%–5.8% EtOAc/hexanes) as a colorless solid (51.3 mg, 0.400 mmol, 80% yield).

*4-Chlorobenzonitrile* (**2c**) [46,56]: Following the general procedure **A**, a solution of 4-chlorobenzaldehyde **1c** (70.3 mg, 0.500 mmol, 1.0 equiv) and TMSN_3_ (115 mg, 1.00 mmol, 2.0 equiv) in HFIP/ACN mixture (2.0 mL, 1:1) was treated with TfOH (17.7 μL, 0.200 mmol, 0.40 equiv). The reaction mixture was stirred at rt for 45 min. Purification using a 4 g silica flash column on a CombiFlash purification system (0%–5% EtOAc/hexanes over 50 min) afforded **2c** (eluted between 0%–0.5% EtOAc/hexanes) as a colorless solid (41.8 mg, 0.304 mmol, 61% yield).

*Methyl 4-cyanobenzoate* (**2d**) [46]: Following the general procedure **A**, a solution of methyl 4-formylbenzoate **1d** (82.1 mg, 0.500 mmol, 1.0 equiv) and TMSN_3_ (115 mg, 1.00 mmol, 2.0 equiv) in HFIP/ACN mixture (2.0 mL, 1:1) was treated with TfOH (17.7 μL, 0.200 mmol, 0.40 equiv). The reaction mixture was stirred at rt for 30 min. Purification using a 4 g silica flash column on a CombiFlash purification system (0%–10% EtOAc/hexanes over 40 min) afforded **2d** (eluted between 2.5%–4.2% EtOAc/hexanes) as a colorless crystalline solid (63.0 mg, 0.391 mmol, 78% yield).

*4-Methylsulfonylbenzonitrile* (**2e**) [57]: Following a slight modification of the general procedure **A**, a solution of 4-methylsulfonylbenzaldehyde **1e** (92.1 mg, 0.500 mmol, 1.0 equiv) and TMSN_3_ (115 mg, 1.00 mmol, 2.0 equiv) in HFIP/ACN mixture (2.0 mL, 1:1) was treated with TfOH (26.6 μL, 0.300 mmol, 0.60 equiv). The reaction mixture was stirred at rt for 45 min. Purification using a 4 g silica flash column on a CombiFlash purification system (0%–40% EtOAc/hexanes over 40 min) afforded **2e** (eluted between 25%–35% EtOAc/hexanes) as a colorless solid (72.9 mg, 0.402 mmol, 81% yield).

*4-Hydroxybenzonitrile* (**2f**) [46]: Following the general procedure **A**, a solution of 4-hydroxybenzaldehyde **1f** (61.1 mg, 0.500 mmol, 1.0 equiv) and TMSN_3_ (115 mg, 1.00 mmol, 2.0 equiv) in HFIP/ACN mixture (2.0 mL, 1:1) was treated with TfOH (17.7 μL, 0.200 mmol, 0.40 equiv). The reaction mixture was stirred at rt for 30 min. Purification using a 4 g silica flash column on a CombiFlash purification system (0%–30% EtOAc/hexanes over 40 min) afforded **2f** (eluted between 15%–20% EtOAc/hexanes) as a colorless crystalline solid (56.5 mg, 0.474 mmol, 95% yield). 

*4-Methoxybenzonitrile* (**2g**) [46]: Following the general procedure **A**, a solution of *p*-anisaldehyde **1g** (68.1 mg, 0.500 mmol, 1.0 equiv) and TMSN_3_ (115 mg, 1.00 mmol, 2.0 equiv) in HFIP/ACN mixture (2.0 mL, 1:1) was treated with TfOH (17.7 μL, 0.200 mmol, 0.40 equiv). The reaction mixture was stirred at rt for 30 min. Purification using a 4 g silica flash column on a CombiFlash purification system (0%–10% EtOAc/hexanes over 40 min) afforded **2g** (eluted between 2.3%–3.2% EtOAc/hexanes) as a colorless crystalline solid (54.4 mg, 0.409 mmol, 82% yield). 

*4-Butoxybenzonitrile* (**2h**) [15]: Following the general procedure **A**, a solution of 4-butoxybenzaldehyde **1h** (89.1 mg, 0.500 mmol, 1.0 equiv) and TMSN_3_ (115 mg, 1.00 mmol, 2.0 equiv) in HFIP/ACN mixture (2.0 mL, 1:1) was treated with TfOH (17.7 μL, 0.200 mmol, 0.40 equiv). The reaction mixture was stirred at rt for 30 min. Purification using a 4 g silica flash column on a CombiFlash purification system (0%–5% EtOAc/hexanes over 40 min) afforded **2h** (eluted between 1.1%–1.8% EtOAc/hexanes) as a colorless oil (71.9 mg, 0.410 mmol, 82% yield). 

*4-(Benzyloxy)benzonitrile* (**2i**) [46]: Following the general procedure **A**, a solution of 4-(benzyloxy)benzaldehyde **1i** (106 mg, 0.500 mmol, 1.0 equiv) and TMSN_3_ (115 mg, 1.00 mmol, 2.0 equiv) in HFIP/ACN mixture (2.0 mL, 1:1) was treated with TfOH (17.7 μL, 0.200 mmol, 0.40 equiv). The reaction mixture was stirred at rt for 20 min. Purification using a 4 g silica flash column on a CombiFlash purification system (0%–10% EtOAc/hexanes over 40 min) afforded **2i** (eluted between 2.3%–3.2% EtOAc/hexanes) as a colorless crystalline solid (74.1 mg, 0.354 mmol, 71% yield).

*4-(Allyloxy)benzonitrile* (**2j**) [46]: Following the general procedure **A**, a solution of 4-(allyloxy)benzaldehyde **1j** ( 81.1 mg, 0.500 mmol, 1.0 equiv) and TMSN_3_ (115 mg, 1.00 mmol, 2.0 equiv) in HFIP/ACN mixture (2.0 mL, 1:1) was treated with TfOH (17.7 μL, 0.200 mmol, 0.40 equiv). The reaction mixture was stirred at rt for 45 min. Purification using a 4 g silica flash column on a CombiFlash purification system (0%–10% EtOAc/hexanes over 50 min) afforded **2j** (eluted between 3.0%–4.0% EtOAc/hexanes) as a colorless solid (71.4mg, 0.448 mmol, 90% yield). 

*4-(Prop-2-yn-1-yloxy)benzonitrile* (**2k**) [46]: Following the general procedure **A**, a solution of 4-(prop-2-yn-1-yloxy)benzaldehyde **1k** (80.1 mg, 0.500 mmol, 1.0 equiv) and TMSN_3_ (115 mg, 1.00 mmol, 2.0 equiv) in HFIP/ACN mixture (2.0 mL, 1:1) was treated with TfOH (17.7 μL, 0.200 mmol, 0.40 equiv). The reaction mixture was stirred at rt for 30 min. Purification using a 4 g silica flash column on a CombiFlash purification system (0%–10% EtOAc/hexanes over 40 min) afforded **2k** (eluted between 3.8%–5.0% EtOAc/hexanes) as a colorless solid (51.4mg, 0.327 mmol, 65% yield).

*4-(Methylthio)benonitrile* (**2l**) [58]: Following the general procedure **A**, a solution of 4-(methylthio)benzaldehyde **1l** (76.2 mg, 0.500 mmol, 1.0 equiv) and TMSN_3_ (115 mg, 1.00 mmol, 2.0 equiv) in HFIP/ACN mixture (2.0 mL, 1:1) was treated with TfOH (17.7 μL, 0.200 mmol, 0.40 equiv). The reaction mixture was stirred at rt for 30 min. Purification using a 4 g silica flash column on a CombiFlash purification system (0%–10% EtOAc/hexanes over 40 min) afforded **2l** (eluted between 4.0%–4.5% EtOAc/hexanes) as a colorless solid (67.2 mg, 0.450 mmol, 90% yield). 

*4-(4-Morpholinyl)benzonitrile* (**2m**) [59]: Following the general procedure **A**, a solution of 4-(4-morpholinyl)benzaldehyde **1m** (95.6 mg, 0.500 mmol, 1.0 equiv) and TMSN_3_ (115 mg, 1.00 mmol, 2.0 equiv) in HFIP/ACN mixture (2.0 mL, 1:1) was treated with TfOH (61.9 μL, 0.700 mmol, 1.40 equiv). The reaction mixture was stirred at rt for 60 min. Purification using a 4 g silica flash column on a CombiFlash purification system (0%–1.5% MeOH/DCM over 40 min) afforded **2m** (eluted between 0.4%–0.8% MeOH/DCM) as a light yellow solid (79.8 mg, 0.424 mmol, 85% yield). 

*4-tert-Butylbenzonitrile* (**2n**) [56]: Following the general procedure **A**, a solution of 4-*tert*-butylbenzaldehyde **1n** (81.1 mg, 0.500 mmol, 1.0 equiv) and TMSN_3_ (115 mg, 1.00 mmol, 2.0 equiv) in HFIP/ACN mixture (2.0 mL, 1:1) was treated with TfOH (17.7 μL, 0.200 mmol, 0.40 equiv). The reaction mixture was stirred at rt for 30 min. Purification using a 4 g silica flash column on a CombiFlash purification system (0%–10% EtOAc/hexanes over 40 min) afforded **2n** (eluted between 0%–1% EtOAc/hexanes) as a yellow oil (56.9 mg, 0.357 mmol, 72% yield). 

*Biphenyl-4-carbonitrile* (**2o**) [46]: Following the general procedure **A**, a solution of biphenyl-4-carboxaldehyde **1o** (91.1 mg, 0.500 mmol, 1.0 equiv) and TMSN_3_ (115 mg, 1.00 mmol, 2.0 equiv) in HFIP and ACN mixture (2.0 mL, 1:1) was treated with TfOH (17.7 μL, 0.200 mmol, 0.40 equiv). The reaction mixture was stirred at rt for 45 min. Purification using a 4 g silica flash column on a CombiFlash purification system (0%–5% EtOAc/hexanes over 50 min) afforded **2o** (eluted between 0%–1.5% EtOAc/hexanes) as an off-white solid (71.4 mg, 0.398 mmol, 80% yield).

*3-Ethoxybenzonitrile* (**2p**) [55]: Following the general procedure **A**, a solution of 3-ethoxybenzaldehyde **1p** (75.1 mg, 0.500 mmol, 1.0 equiv) and TMSN_3_ (115 mg, 1.00 mmol, 2.0 equiv) in HFIP/ACN mixture (2.0 mL, 1:1) was treated with TfOH (17.7 μL, 0.200 mmol, 0.40 equiv). The reaction mixture was stirred at rt for 60 min. Purification using a 4 g silica flash column on a CombiFlash purification system (0%–5% EtOAc/hexanes over 50 min) afforded **2p** (eluted between 1.2%–1.5% EtOAc/hexanes) as a colorless oil (44.0 mg, 0.299 mmol, 60% yield).

*2-Methoxybenzonitrile* (**2q**) [55]: Following the general procedure **A**, a solution of *o*-anisaldehyde **1q** (68.1 mg, 0.500 mmol, 1.0 equiv) and TMSN_3_ (115 mg, 1.00 mmol, 2.0 equiv) in HFIP/ACN mixture (2.0 mL, 1:1) was treated with TfOH (17.7 μL, 0.200 mmol, 0.40 equiv). The reaction mixture was stirred at rt for 60 min. Purification using a 4 g silica flash column on a CombiFlash purification system (0%–10% EtOAc/hexanes over 40 min) afforded **2q** (eluted between 2.5%–5.0% EtOAc/hexanes) as a colorless oil (46.4 mg, 0.348 mmol, 70% yield). 

*2-Bromobenzonitrile* (**2r**) [46]: Following the general procedure **A**, a solution of 2-bromobenzaldehyde **1r** (92.5 mg, 0.500 mmol, 1.0 equiv) and TMSN_3_ (115 mg, 1.00 mmol, 2.0 equiv) in HFIP/ACN mixture (2.0 mL, 1:1) was treated with TfOH (17.7 μL, 0.200 mmol, 0.40 equiv). The reaction mixture was stirred at rt for 30 min. Purification using a 4 g silica flash column on a CombiFlash purification system (0%–10% EtOAc/hexanes over 40 min) afforded **2r** (eluted between 2.0%–2.5% EtOAc/hexanes) as a colorless crystalline solid (61.7 mg, 0.339 mmol, 68% yield).

*1,3-Benzodioxole-5-carbonitrile* (**2s**) [46]: Following the general procedure **A**, a solution of piperonal **1s** (75.1 mg, 0.500 mmol, 1.0 equiv) and TMSN_3_ (115 mg, 1.00 mmol, 2.0 equiv) in HFIP/ACN mixture (2.0 mL, 1:1) was treated with TfOH (17.7 μL, 0.200 mmol, 0.40 equiv). The reaction mixture was stirred at rt for 60 min. Purification using a 4 g silica flash column on a CombiFlash purification system (0%–25% EtOAc/hexanes over 40 min) afforded **2s** (eluted between 3.8%–5.6% EtOAc/hexanes) as a colorless solid (64.8 mg, 0.441 mmol, 88%). 

*3,4-Dimethoxybenzonitrile* (**2t**) [46]: Following the general procedure **A**, a solution of 3,4-dimethoxybenzaldehyde **1t** (83.1 mg, 0.500 mmol, 1.0 equiv) and TMSN_3_ (115 mg, 1.00 mmol, 2.0 equiv) in HFIP/ACN mixture (2.0 mL, 1:1) was treated with TfOH (17.7 μL, 0.200 mmol, 0.40 equiv). The reaction mixture was stirred at rt for 20 min. Purification using a 4 g silica flash column on a CombiFlash purification system (0%–30% EtOAc/hexanes over 40 min) afforded **2t** (eluted between 11%–16% EtOAc/hexanes) as a colorless crystalline solid (70.0 mg, 0.429 mmol, 86% yield). 

*4-Hydroxy-3-methoxybenzonitrile* (**2u**) [60,61]: Following the general procedure **A**, a solution of 4-hydroxy-3-methoxybenzaldehyde (vanillin) **1u** (76.1 mg, 0.500 mmol, 1.0 equiv) and TMSN_3_ (115 mg, 1.00 mmol, 2.0 equiv) in HFIP/ACN mixture (2.0 mL, 1:1) was treated with TfOH (17.7 μL, 0.200 mmol, 0.40 equiv). The reaction mixture was stirred at rt for 30 min. Purification using a 4 g silica flash column on a CombiFlash purification system (0%–25% EtOAc/hexanes over 50 min) afforded **2u** (eluted between 12.5%–16% EtOAc/hexanes) as a colorless crystalline solid (66.5 mg, 0.446 mmol, 89% yield).

*3-Ethoxy-4-hydroxybenzonitrile* (**2v**) [62]: Following the general procedure **A**, a solution of 3-ethoxy-4-hydroxybenzaldehyde **1v** (83.1 mg, 0.500 mmol, 1.0 equiv) and TMSN_3_ (115 mg, 1.00 mmol, 2.0 equiv) in HFIP/ACN mixture (2.0 mL, 1:1) was treated with TfOH (17.7 μL, 0.200 mmol, 0.40 equiv). The reaction mixture was stirred at rt for 75 min. Purification using a 4 g silica flash column on a CombiFlash purification system (0%–25% EtOAc/hexanes over 40 min) afforded **2v** (eluted between 8.1%–12.5% EtOAc/hexanes) as a colorless solid (74.3 mg, 0.455 mmol, 91% yield). 

*4-Hydroxy-3-nitrobenzonitrile* (**2w**) [46]: Following the general procedure **A**, a solution of 4-hydroxy-3-nitrobenzaldehyde **1w** (83.6 mg, 0.500 mmol, 1.0 equiv) and TMSN_3_ (115 mg, 1.00 mmol, 2.0 equiv) in HFIP/ACN mixture (2.0 mL, 1:1) was treated with TfOH (17.7 μL, 0.200 mmol, 0.40 equiv). The reaction mixture was stirred at rt for 45 min. Purification using a 4 g silica flash column on a CombiFlash purification system (0%–20% EtOAc/hexanes over 40 min) afforded **2w** (eluted between 6.5%–9% EtOAc/hexanes) as a yellow solid (67.7 mg, 0.413 mmol, 82% yield). 

*4-Hydroxy-(1,1-biphenyl)-3-carbonitrile* (**2x**) [46]: Following the general procedure **A**, a solution of 4-hydroxy-(1,1-biphenyl)-3-carbaldehyde **1x** (99.1 mg, 0.500 mmol, 1.0 equiv) and TMSN_3_ (115 mg, 1.00 mmol, 2.0 equiv) in HFIP/ACN mixture (2.0 mL, 1:1) was treated with TfOH (17.7 μL, 0.200 mmol, 0.40 equiv). The reaction mixture was stirred at rt for 20 min. Purification using a 4 g silica flash column on a CombiFlash purification system (0%–5% EtOAc/hexanes over 40 min) afforded **2x** (eluted between 1.0%–2.0% EtOAc/hexanes) as a yellow solid (52.5 mg, 0.269 mmol, 54% yield). 

*3,4-Dibromobenzonitrile* (**2y**) [63]: Following the general procedure **A**, a solution of 3,4-dibromobenzaldehyde **1y** (132 mg, 0.500 mmol, 1.0 equiv) and TMSN_3_ (115 mg, 1.00 mmol, 2.0 equiv) in HFIP/ACN mixture (2.0 mL, 1:1) was treated with TfOH (17.7 μL, 0.200 mmol, 0.40 equiv). The reaction mixture was stirred at rt for 45 min. Purification using a 4 g silica flash column on a CombiFlash purification system (100% hexanes over 5 min) afforded **2y** as a colorless solid (108 mg, 0.414 mmol, 83% yield). Mp: 118–120 °C; TLC (10% EtOAc/hexanes): R*_f_* = 0.55; IR (neat) 2227 cm^−1^; ^1^H-NMR (400 MHz, CDCl_3_) δ 7.88 (d, *J* = 1.9 Hz, 1H), 7.74 (d, *J* = 8.3 Hz, 1H), 7.44 (dd, *J* = 8.3, 1.9 Hz, 1H); ^13^C-NMR (101 MHz, CDCl_3_) δ 136.8, 134.7, 131.6, 131.0, 126.1, 116.8, 112.9. Compound **2y** did not afford a good parent ion in MS.

*2-Naphthonitrile* (**2z**) [55]: Following the general procedure **A**, a solution of 2-naphthaldehyde **1z** (78.1 mg, 0.500 mmol, 1.0 equiv) and TMSN_3_ (115 mg, 1.00 mmol, 2.0 equiv) in HFIP/ACN mixture (2.0 mL, 1:1) was treated with TfOH (17.7 μL, 0.200 mmol, 0.40 equiv). The reaction mixture was stirred at rt for 60 min. Purification using a 4 g silica flash column on a CombiFlash purification system (0%–10% EtOAc/hexanes over 50 min) afforded **2z** (eluted between 0.1%–0.4% EtOAc/hexanes) as a light yellow solid (59.0 mg, 0.385 mmol, 77% yield).

*Benzofuran-2-carbonitrile* (**2aa**) [64]: Following the general procedure **A**, a solution of 2-benzofurancarboxaldehyde **1aa** (73.1 mg, 0.500 mmol, 1.0 equiv) and TMSN_3_ (115 mg, 1.00 mmol, 2.0 equiv) in HFIP/ACN mixture (2.0 mL, 1:1) was treated with TfOH (17.7 μL, 0.200 mmol, 0.40 equiv). The reaction mixture was stirred at rt for 20 min. Purification using a 4 g silica flash column on a CombiFlash purification system (0%–10% EtOAc/hexanes over 40 min) afforded **2aa** (eluted between 0.5%–1.8% EtOAc/hexanes) as a yellow solid (55.2 mg, 0.386 mmol, 77% yield). 

*Benzo[b]thiophene-3-carbonitrile* (**2ab**) [65]: Following the general procedure **A**, a solution of thianaphthene-3-carboxaldehyde **1ab** (81.1 mg, 0.500 mmol, 1.0 equiv) and TMSN_3_ (115 mg, 1.00 mmol, 2.0 equiv) in HFIP/ACN mixture (2.0 mL, 1:1) was treated with TfOH (17.7 μL, 0.200 mmol, 0.40 equiv). The reaction mixture was stirred at rt for 20 min. Purification using a 4 g silica flash column on a CombiFlash purification system (0%–5% EtOAc/hexanes over 50 min) afforded **2ab** (eluted between 0.4%–0.9% EtOAc/hexanes) as a colorless crystalline solid (43.8 mg, 0.275 mmol, 55% yield).

*Cinnamonitrile* (**2ac**) [46]: Following the general procedure **A**, a solution of *trans*-cinnamaldehyde **1ac** (66.1 mg, 0.500 mmol, 1.0 equiv) and TMSN_3_ (115 mg, 1.00 mmol, 2.0 equiv) in HFIP/ACN mixture (2.0 mL, 1:1) was treated with TfOH (17.7 μL, 0.200 mmol, 0.40 equiv). The reaction mixture was stirred at rt for 45 min. Purification using a 4 g silica flash column on a CombiFlash purification system (0%–10% EtOAc/hexanes over 40 min) afforded **2ac** (eluted between 2.3%–2.8% EtOAc/hexanes) as a colorless oil (58.0 mg, 0.449 mmol, 90% yield).

*(E)-3-(4-Methoxyphenyl)acrylonitrile* (**2ad**) [66]: Following the general procedure **A**, a solution of 4-methoxycinnamaldehyde **1ad** (81.1 mg, 0.500 mmol, 1.0 equiv) and TMSN_3_ (115 mg, 1.00 mmol, 2.0 equiv) in HFIP/ACN mixture (2.0 mL, 1:1) was treated with TfOH (17.7 μL, 0.200 mmol, 0.40 equiv). The reaction mixture was stirred at rt for 20 min. Purification using a 4 g silica flash column on a CombiFlash purification system (0%–10% EtOAc/hexanes over 40 min) afforded **2ad** (eluted between 4.3%–5.5% EtOAc/hexanes) as a colorless solid (73.1 mg, 0.459 mmol, 92% yield). 

*α-Methyl-trans-cinnamonitrile* (**2ae**) [67,68]: Following the general procedure **A**, a solution of *α*-methyl-*trans*-cinnamaldehyde **1ae** (73.1 mg, 0.500 mmol, 1.0 equiv) and TMSN_3_ (115 mg, 1.00 mmol, 2.0 equiv) in HFIP/ACN mixture (2.0 mL, 1:1) was treated with TfOH (17.7 μL, 0.200 mmol, 0.40 equiv). The reaction mixture was stirred at rt for 45 min. Purification using a 12 g silica flash column on a CombiFlash purification system (0%–5% EtOAc/hexanes over 50 min) afforded **2ae** (eluted between 0.5%–1.5% EtOAc/hexanes) as a pale yellow oil (38.0 mg, 0.265 mmol, 53% yield).

*3-Phenylpropionitrile* (**2af**) [69,70]: Following a slight modification of the general procedure **A**, a solution of *ca.* 77% pure hydrocinnamaldehyde **1af** (26.8 mg, 0.200 mmol, 1.0 equiv; uncorrected for impurities) and TMSN_3_ (69.1 mg, 0.600 mmol, 3.0 equiv) in HFIP/ACN mixture (1.0 mL, 1:1) was treated with TfOH (4.43 μL, 0.0500 mmol, 0.25 equiv). The reaction mixture was stirred at rt for 60 min. Purification using a 4 g silica flash column on a CombiFlash purification system (0%–5% EtOAc/hexanes over 40 min) afforded **2af** (eluted between 2.8–3.4% EtOAc/hexanes) as a colorless oil (8.00 mg, 0.0610 mmol, 30% uncorrected yield and *ca.* 40% corrected yield w.r.t. 77% purity of **1af**).
